# Pathological Changes of the Anterior Lens Capsule

**DOI:** 10.1155/2021/9951032

**Published:** 2021-05-04

**Authors:** Wei Liu, Dandan Huang, Ruru Guo, Jian Ji

**Affiliations:** ^1^Tianjin Key Laboratory of Retinal Functions and Diseases, Tianjin Branch of National Clinical Research Center for Ocular Disease, Eye Institute and School of Optometry, Tianjin Medical University Eye Hospital, Tianjin 300384, China; ^2^Department of Ophthalmology, University of Groningen, University Medical Center Groningen, Groningen, Netherlands; ^3^Department of Ophthalmology, Taihe Hospital, Hubei University of Medicine, Shiyan, Hubei, China

## Abstract

The anterior lens capsule (ALC), as the thickest basement membrane in the body, is an acellular, soft, smooth, transparent membrane secreted by lens epithelial cells. The ALC has its unique biomechanical properties to serve as a barrier and separate the lens from infectious viruses and bacteria together with the posterior capsule and pericapsular membrane. However, the biomechanical and ultrastructural properties of the ALC can be changed under certain conditions. Here, we provide a brief review of the pathological changes of the ALC in several eye disorders, including cataract, aniridia, climatic droplet keratopathy, exfoliation syndrome, true exfoliation syndrome, Alport syndrome, and silicone oil tamponade.

## 1. Introduction

The anterior lens capsule (ALC), as the thickest basement membrane in the body, encapsulates the crystalline lens and acts as a barrier and separates the lens from infectious viruses and bacteria together with the posterior capsule and pericapsular membrane. The ALC is an acellular, soft, smooth, transparent basement membrane secreted by lens epithelial cells (LECs). The ALC with the accompanying monolayer subcapsular epithelium represents the most important metabolic element of the crystalline lens. Recently, the biomechanical properties and biomedical engineering perspectives of the ALC were reviewed by us [1]. Also, here, we provide a brief review of the pathological changes of the ALC in several eye disorders, which were never mentioned previously.

## 2. Pathological Changes

### 2.1. Cataract

Cataract is most simply defined as opacification of the crystalline lens inside the eye, which is the commonest cause of vision loss worldwide [2, 3]. Under normal physiological conditions, the ALC with the lens epithelium represents a major site of ion transport and fluid transport, which plays a vital role in maintaining lens homeostasis and transparency by providing the driving force for the ionic gradients and the fluid circulation [4, 5]. Therefore, any factor disturbing the transport processes, components or biomechanical characteristics of the ALC, and morphology or biochemistry of the lens epithelium will lead to water accumulation in the lens and the subsequent unbalance of lens homeostasis, thus resulting in cataract formation [6]. Using AFM, Choi et al. [7] found that the cataract group showed significantly lower surface roughness in the anterior side of the ALC and higher surface roughness in their posterior side than the noncataract control group. They also found lower Young's modulus in the cataract group compared to the control group, regardless of the ALC side. Compared to nuclear cataracts, intumescent white cataracts do not have a significant difference in ALC thickness but differ in ultrastructure morphology, including extrusions at the basement membrane epithelial border, lamellation, rarefication, and filaments in the basement membrane [8].

In eyes with mature cataracts, poor fundus red reflex and poor visibility of the capsule makes surgery more challenging. Therefore, capsule staining is often performed to enable the round edges of the capsulorhexis visible and facilitate the continuous curvilinear capsulorhexis (CCC) procedure. Several studies have verified that biomechanical properties of the ALC would change after vital dyes staining, including trypan blue, brilliant blue, and indocyanine green [9–13]. The darker and longer capsule staining that trypan blue provides is particularly advantageous, making it preferable to other agents for dye-aided cataract surgery. It has been reported that the stained capsules in human led to a decrease in elasticity and an increase in stiffness, especially in diabetic patients [13]. This effect is probably a light-dependent process and resulted from the photosensitizing action of trypan blue and light-induced collagen crosslinking of the capsule collagen because no biomechanical changes were found in porcine capsules with trypan blue staining in the absence of light or after a short illumination time of 30 seconds [12]. However, it is also reported that trypan blue application had no effect on capsule elasticity and stiffness [14]. By using a mechanized tensile strength model, Jaber et al. [15] found that there was no difference in CCC strength between trypan-blue-stained capsules and control capsules, indicating staining with trypan blue did not reduce CCC tear resistance.

### 2.2. Aniridia

Aniridia is characterized by underdeveloped iris and accompanied by abnormalities of the cornea, anterior chamber angle, lens, retina, and optic nerve [16, 17]. It has been discovered that the ALC from some aniridic patients is thinner and more fragile, making the capsulorhexis more challenging [17, 18]. Degenerative changes (degeneration, necrosis, and loss) and proliferative changes (proliferation and double layer) of the lens epithelium were also reported in familiar aniridia with cataract [19]. The exact cause of the thin, friable ALC in aniridia is unknown; one possibility is the absence of or diminished content in one or more of the ALC constituents.

### 2.3. Climatic Droplet Keratopathy

Climatic droplet keratopathy (CDK) is a corneal degeneration disease and characterized by a band‐shaped pattern of subepithelial opacities and golden‐yellow spherules [20, 21]. There is a strong association between changes of the ALC and presence of CDK. The capsule changes are usually confined to the central pupillary area, which includes a white opalescence, an elevation in front of the contour of the rest of the lens to form a plateau, and a “bag” or herniation of the lens capsule through the pupil. These ALC changes might be caused by excessive ultraviolet light exposure, which is also the main cause of CDK [22].

### 2.4. Exfoliation Syndrome

Exfoliation syndrome (XFS) or pseudoexfoliation syndrome is an age-related disease in which abnormal fibrillar extracellular material is produced and accumulates in many ocular tissues, mainly the ALC and the pupillary margin. The typical distribution of ALC deposits consists of three zones: a granular, often layered, peripheral zone; a central disc, and a clear area between them. Several studies confirmed fibrils accumulation above or in the basement membrane of the ALC in XFS eyes [23–28], and another unknown, electron-dense, microgranular, unbound material was also observed by transmission electron microscopy beneath the lens epithelium in XFS patients [29]. By immunofluorescence and electron microscopic immunogold techniques, heparan sulfate and chondroitin sulfate proteoglycans, laminin, entactin/nidogen, fibronectin, and amyloid P protein were shown to be an integral constituent of XFS material [30]. The ALC thickness was reported to vary greatly measured by light microscopy, and there was no statistical difference between XFS lenses and controls [31], while with high-resolution anterior segment optical coherence tomography, the ALC was found to be thicker in XFS patients than normal people [32]. The ALC ultrastructural abnormalities (diffuse intracellular and extracelluar edema, transparent vacuoles, apoptotic cells, and destroyed epithelial cells) were also found to be more extended and more frequently observed in XFS patients than cataract controls [33]. XFS can be unilateral or bilateral, but exfoliation material can also be found in the unaffected eyes of patients with clinically unilateral XFS [34, 35]. If capsule staining (i.e., trypan blue) is needed, lower concentration and/or exposure is recommended because the ALC has more affinity to trypan blue in XFS patients [36].

### 2.5. True Exfoliation Syndrome

True exfoliation syndrome (TEX) is a rare disorder in which characteristic lamellar separation of the ALC occurs. The pathogenesis of TEX is not clear; although intense infrared radiation is thought to be the main causative factor, most cases are idiopathic. Histologically, a thickened delaminated structure, perpendicular fibrils and vesicular degeneration in the capsule, and degenerative lens epithelium have been documented [37]. Recently, double delamination and pigment deposition on the detached membrane are reported to be new findings in TEX patients [38].

### 2.6. Alport Syndrome

Alport syndrome is a rare disorder of the basement membrane characterized clinically by progressive hereditary nephritis, sensorineural hearing loss, and ocular abnormalities. Genetically, Alport syndrome is due to mutations involving the coding for type IV collagen resulting in a defective synthesis of type IV collagen [39]. Clinically, the typical ocular manifestations of Alport syndrome are a flecked retinopathy and bilateral anterior lenticonus, which is resulted from the conical protrusion of the lens anteriorly through the thinnest and weakest part of the capsule [39]. Several electron microscopic studies have demonstrated the marked thinning and vertical dehiscence of ALC in Alport syndrome [40–43]. Spontaneous rupture of the ALC was also reported, which is suggestive of defective capsular strength [44, 45]. By using the lens capsule of wild-type and Alport syndrome mice as a model, the osmotic swelling experiments from the work of Gyoneva et al. [46] revealed direction-dependent changes. They found Alport lenses strained significantly more than wild-type lenses in the anterior-posterior direction, which is consistent with clinical data: Alport patients develop conical protrusions on the anterior and posterior lenticular poles.

### 2.7. Silicone Oil Tamponade

Silicone oil is an intraocular tamponade after vitrectomy surgery, which is used for the treatment of complicated retinal detachment [47]. However, intravitreal silicone oil can lead to several complications including cataract, glaucoma, band keratopathy, oil emulsification [48, 49], and ALC changes.

In Citirik et al.'s study, by electron microscopy, silicone oil was detected on the posterior surface of the ALC in 50% cases and surface irregularities, pits, and depressions were present in the posterior surface of the ALC in all the ten silicone oil tamponade cases [50]. Ultrastructural effects of silicone oil on the ALC of the clear crystalline lens of myopic eyes were also studied [51]. Light microscopic examination showed relatively more flat cells with irregular outline of LECs with wide intercellular spaces, deeply stained nuclei, and multiple intracytoplasmic vacuoles. Collagenous surfaces filled with multiple pits, depressions, and abnormal deposits were found under scanning electron microscopy, while transmission electron microscopy revealed LECs with apoptotic changes, cytoplasmic vacuoles, and filopodia-like protrusions between LECs and the capsule [51]. In the ALC of rabbit eyes with silicone oil tamponade, many vacuoles amid matrix accumulation were present on the posterior surface, suggesting the deposition of emulsified silicone oil droplets [52], which is similar to the histopathological findings of human eyes [53].

Clinically, rigidity of the ALC is frequently encountered during cataract surgery in silicone-oil-filled eyes [54], which increases the mechanical difficulties of anterior capsulorrhexis. The anterior subcapsular tissue plaque resulted from silicone oil tamponade may be responsible for the increased mechanical resistance of the ALC [55].

The different pathological changes of the ALC in different diseases are summarized in Table 1.

## 3. Conclusions

The ALC, as structural support for the lens within the eye, plays an important role on normal lens growth and metabolism. However, the biomechanical properties of the ALC may change in several ocular diseases, including cataract, aniridia, climatic droplet keratopathy, exfoliation syndrome, true exfoliation syndrome, Alport syndrome, and silicone oil tamponade. These pathological changes vary from biomechanical alterations (surface roughness, Young's modulus, elasticity, stiffness, rigidity, fragility, etc.) to ultrastructural abnormalities (increase or decrease in thickness, abnormal material accumulation, lamellar separation, vesicular degeneration, ALC dehiscence, surface irregularities, cytoplasmic vacuoles, etc.) in different ocular diseases. If cataract surgery is scheduled for these eyes, the surgery procedure, especially the capsulorhexis, would be challenging. Therefore, attention should be raised when performing cataract surgery for these patients.

## Figures and Tables

**Table 1:** Pathological changes of the ALC in different diseases.

Disease	Main changes
Cataract	Lower surface roughness in the anterior side of the ALC and higher surface roughness in their posterior side
	Lower Young's modulus
	Ultrastructure morphology changes in intumescent white cataracts
Trypan blue staining	Decrease in elasticity and an increase in stiffness
	No difference in CCC strength
Aniridia	Thinner and more fragile
	Degenerative changes (degeneration, necrosis, and loss) and proliferative changes (proliferation and double layer) of the lens epithelium
Climatic droplet keratopathy	Plateau or “bag” or herniation-like of the lens capsule
Exfoliation syndrome	Abnormal fibrillar extracellular material deposition
	Diffuse intracellular and extracelluar edema, transparent vacuoles, apoptotic cells, and destroyed epithelial cells
	More affinity to trypan blue
True exfoliation syndrome	Thickened delaminated structure, perpendicular fibrils, and vesicular degeneration in the ALC
	Double delamination and pigment deposition on the detached membrane
Alport syndrome	Marked thinning and vertical dehiscence of the ALC
	Spontaneous rupture of the ALC
Silicone oil tamponade	Surface irregularities, pits, and depressions in the posterior surface of the ALC
	LECs with apoptotic changes and cytoplasmic vacuoles
	Increased mechanical resistance of the ALC

ALC: anterior lens capsule; CCC: continuous curvilinear capsulorhexis; LECs: lens epithelium cells.

## Data Availability

The datasets used and/or analysed during the current study are available from the corresponding author on reasonable request.
